# Matrix-producing carcinoma of the breast showing retained rim enhancement to the late phase on magnetic resonance imaging

**DOI:** 10.1016/j.radcr.2023.09.084

**Published:** 2023-10-19

**Authors:** Mami Yoshida, Shoji Oura

**Affiliations:** Department of Surgery, Kishiwada Tokushukai Hospital, Kishiwada-City, Japan

**Keywords:** Breast cancer, Hypo-intensity on T2-weighted image, Matrix-producing carcinoma, Retained rim enhancement

## Abstract

A 54-year-old woman with a left breast mass was referred to our hospital. Mammography showed a mass, 2.1cm in size, with micro-lobulated boarders. Ultrasonography showed an oval mass with predominant low internal echoes and enhanced posterior echoes. Core needle biopsy of the tumor showed malignant cells and chondroid matrices. With MRI, the tumor was hypo-intense on T1-weighted images, mixed hyper- and hypo-intense on T2-weighted images, and completely rim enhanced until late phase on time-signal intensity curve. Despite the good indication for breast conserving therapy, patient's preference made her undergo total mastectomy, sentinel lymph node biopsy, and immediate breast reconstruction using an extended latissimus dorsi musculocutaneous flap. Postoperative pathologic study showed large acellular areas, atypical cells growing in cord-like and linear fashions with cartilage-like matrices, and no spindle cells / osteoclasts between the cancer cells and chondroid matrices, leading to the pathologic diagnosis of matrix-producing carcinoma. The patient received dose-dense chemotherapy as an adjuvant therapy and has been well without any recurrences for 14 months. Physicians should note that partial hypo-intensity on T2-weighted images and retained rim enhancement to the late phase should be important findings of breast matrix-producing carcinoma.

## Introduction

Due to the advent of breast-conserving therapy, the presence of ductal spread and ipsilateral multiple lesions, which are negligible factors in mastectomy, has become of great significance on deciding how to operate breast cancer. Magnetic resonance imaging (MRI), therefore, has frequently come to be used to detect such lesions before breast cancer surgery due to its excellent spatial resolution and no X-ray exposure. In addition to the assessment of cancer distribution in the breast, time-signal intensity curve of MRI can suggest the intratumoral pathologic components [1].

Matrix-producing carcinoma is a relatively rare subtype of metaplastic carcinomas and is composed of an admixture of mesenchymal components, that is, chondroid and osteoid component, and carcinomatous components [2]. Because of the similarities of intratumoral components, it sometimes is very difficult to pathologically differentiate matrix-producing carcinoma from carcinoma with osseous/cartilaginous differentiation. The absence of spindle cells and osteoclasts generally brings us to the pathologic diagnosis of matrix-producing carcinoma.

We herein report a case of matrix-producing carcinoma showing partial hypo-intensity on T2-weighted images and retained rim enhancement even in the late phase on time-signal intensity curve.

## Case report

**Fig. 1:**
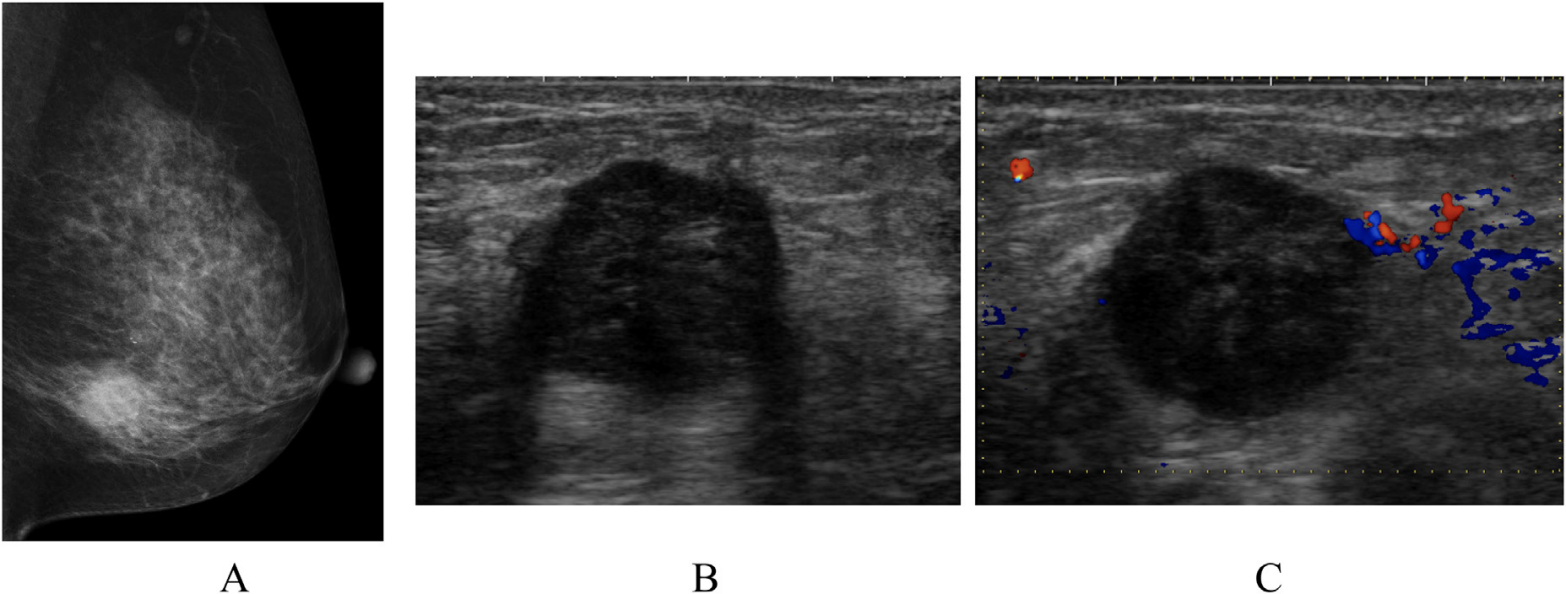
Mammography and ultrasonography. (A) Mammography showed a mass in the lower part of the left breast. (B) Ultrasonography showed a well-circumscribed oval mass with predominantly low internal echoes and enhanced posterior echoes. (C) Doppler ultrasonography showed blood flow just around the tumor.

**Fig. 2:**
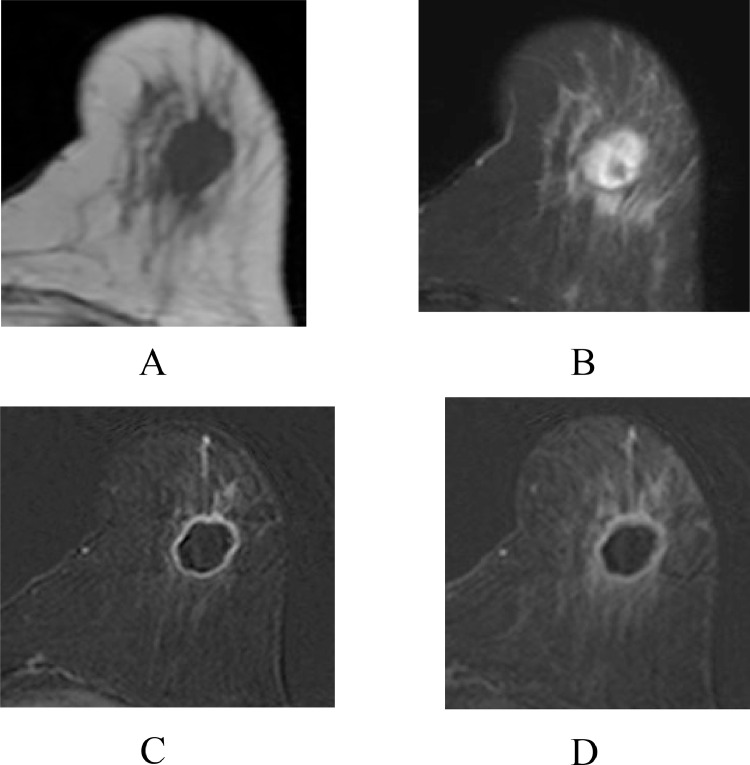
Magnetic resonance imaging (MRI). MRI showed an oval mass with hypo-intensity on T1-weighted images (A), mixed hyper- and hypo-intensity on T2-weighted images (B), and retained rim enhanced until late phase on time-signal intensity curve (C; early phase, D; late phase).

**Fig. 3:**
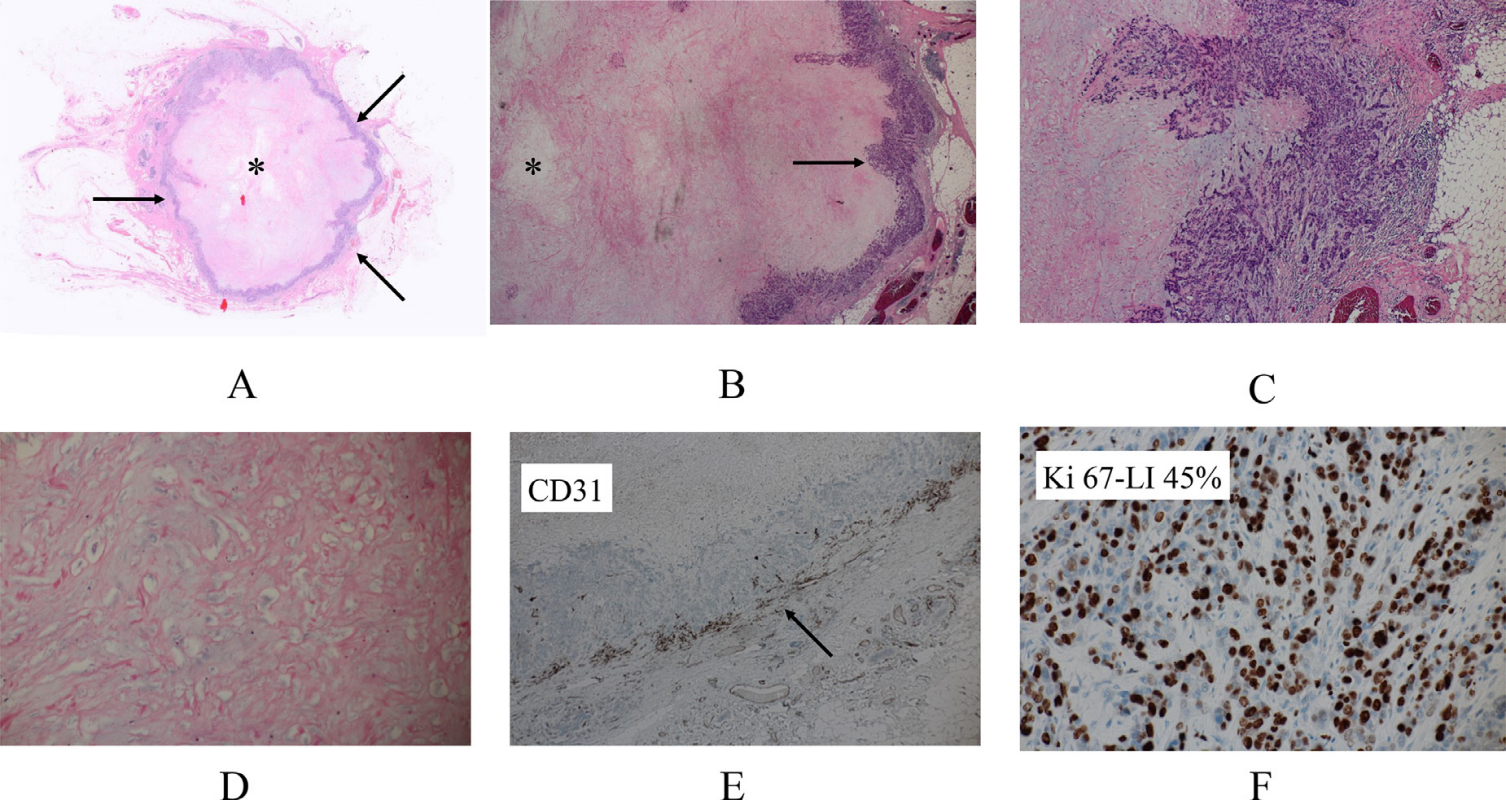
Pathologic findings. (A) Low-magnified view showed an acellular areas (asterisk) encompassed by tumor cells (arrows). (B) Magnified view more clearly showed acellular areas (asterisk) and tumor cells (arrow). (C) Neither spindle cells nor osteoclasts were observed between the tumor cells and the chondroid matrix. (D) Magnified view showed chondroid matrix. (E) CD31 immunostaining showed positivities in the tumor cells but not in the chondroid matrix. (F) Tumor cells showed high Ki-67 labeling index of 45%.

A 54-year-old woman with a left breast mass was referred to our hospital. Mammography showed a mass, 2.1 cm in size, with microlobulated boarders (Fig. 1A). Ultrasonography showed an oval mass with predominant low internal echoes, enhanced posterior echoes, and peritumoral blood flow (Figs. 1B and C). Core needle biopsy of the breast tumor showed extensive acellular areas and triple negative atypical cells growing in cord-like and linear fashions with cartilage-like matrices, highly suggesting matrix-producing carcinoma. With MRI, the tumor was solitary, hypointense on T1-weighted images (Fig. 2A), mixed hyper- and hypointense on T2-weighted images (Fig. 2B), completely rim enhanced even to the late phase on the time-signal intensity curve (Figs. 2C and D), and lacking ductal spread / daughter nodules. All these findings highly suggested the tumor to be well indicated for breast-conserving therapy. The patient, however, detested in-breast recurrence and underwent total glandectomy and sentinel node biopsy followed by immediate breast reconstruction using extended lattisimus dorsi musculocutaneous flap. Postoperative pathologic study showed large acellular areas, atypical cells proliferating with cartilage-like matrices, and no spindle cells/osteoclasts between the cancer cells and chondroid matrices, leading to the pathologic diagnosis of matrix-producing carcinoma (Figs. 3A-D). CD31 immunostaining showed massive presence of vessels in the periphery of the tumor, but scarecely in the center of it (Fig. 3E). Despite the negative sentinel node biopsy, estrogen receptor negativity and high growth potential, that is, Ki-67 labeling index of 45% (Fig. 3F), of the tumor made the patient receive dose-dense chemotherapy as an adjuvant therapy. The patient has been well without any recurrences for 14 months.

## Discussion

Metaplastic carcinomas are defined as the tumors with neoplastic epithelium showing squamous, spindle, chondroid, and osseous differentiations. World Health Organization classifies the metaplastic carcinomas into 5 major subtypes: low grade adenosquamous carcinoma, fibromatosis-like metaplastic carcinoma, spindle cell carcinoma, squamous cell carcinoma, and metaplastic carcinoma with heterologous mesenchymal differentiation [3]. Matrix-producing carcinoma is included into the last subtype.

It has been widely recognized that time-signal intensity curve is very useful to judge the target breast tumor whether to be malignant or benign. Time-signal intensity phenotypes are grossly categorized into 3 types such as washout, plateau, and persistent patterns. A typical washout pattern suggests a cancer-cell-rich mass with high blood flow, highly suggesting a malignant tumor. The plateau and persistent patterns are caused by the considerable presence of various benign pathologic components such as collagen fibers, mucus, and adipocytes in the tumor.

MD Anderson Cancer Center reported a clinicopathologic study of the largest number, that is, 32 cases, of breast matrix-producing carcinomas [2]. Regarding the shape of the matrix-producing carcinoma, they reported that 83% of the cases showed a nodular type. Other reports, ranging from one to several cases, also reported that they all exhibited nodular shapes. The ratio of the matrix in the tumor is 10% or less in 44% of cases and 40% or more in 28% of cases. In addition, 59% of matrix-producing carcinoma cases had a central necrosis pattern, well corresponding to the acellular areas in this case.

Cartilage and bone basically have extremely low protons. They, therefore, generally show low intensity both on T1- and T2-weighted images of MRI. In this case, the tumor had a moderate amount of cartilage matrix in its intratumoral peripheral areas, but also harbored much larger acellular areas, leading to the focal hypo-intense and larger hyper-intense areas on T2-weighted images. On the other hand, clearly judged by CD31 immunostaining, the tumor had very sparse and abundant vessels in the central and peripheral areas of the tumor, respectively. These pathologic findings well matched the partial hypo-intensity on T2-weighted images and retained rim enhancement pattern. In carcinomas with osseous / cartilaginous differentiation, the presence of spindle cells and osteoclasts generally causes a hyper intense pattern in the late phase of the time-signal intensity curve. These image findings should be important discriminants between these 2 disorders.

Many researchers have reported the rim enhancement pattern of matrix-producing carcinoma in the early phase. Of the 12 cases with MRI findings for matrix-producing carcinoma thus far reported [[4], [5], [6], [7]], 11 cases had a rim enhancement pattern, except for one case with a high proportion of cancer cells in the tumor [7]. No authors, however, pointed out the diagnostic importance of the partial hypo-intensity on T2-weighted images and retained rim enhancement pattern to date.

## Conclusion

Clinicians should note that matrix-producing carcinoma is a rare metaplastic carcinoma that shows partial hypo-intensity on T2-weighted images and retained rim enhancement until the late phase due to the paucity of vessels in the central area of the tumor.

## Authors’ contributions

MY designed the concept of this study. SO drafted the manuscript.

## Patient consent

Written informed consent was obtained from the patient for the publication of this case report and any accompanying images.
